# Effects of Shrimp Peptide Hydrolysate on Intestinal Microbiota Restoration and Immune Modulation in Cyclophosphamide-Treated Mice

**DOI:** 10.3390/molecules27051720

**Published:** 2022-03-06

**Authors:** Asif Iqbal Khan, Ata Ur Rehman, Nabeel Ahmed Farooqui, Nimra Zafar Siddiqui, Qamar Ayub, Muhammad Noman Ramzan, Liang Wang, Yi Xin

**Affiliations:** 1 Department of Biotechnology, College of Basic Medical Science, Dalian Medical University, Dalian 116044, China; asif.iqbal@duhs.edu.pk (A.I.K.); ata_burraq@yahoo.com (A.U.R.); nabeel.farooqui99@yahoo.com (N.A.F.); nimra.siddiqui12@gmail.com (N.Z.S.); 2 College of Clinical Laboratory Sciences, Dalian Medical University, Dalian 116044, China; qamar_ayub@yahoo.com; 3 Department of Biochemistry and Molecular Biology, College of Basic Medical Science, Dalian Medical University, Dalian 116044, China; mnomanr8894@gmail.com; 4 Stem Cell Clinical Research Center, National Joint Engineering Laboratory, Regenerative Medicine Center, The First Affiliated Hospital of Dalian Medical University, No. 193, Lianhe Road, Shahekou District, Dalian 116011, China

**Keywords:** shrimp (*Penaeus Chinensis*), peptides, immunosuppression gut microbiota, cyclophosphamide, gut health

## Abstract

The gut microbiota is important in regulating host metabolism, maintaining physiology, and protecting immune homeostasis. Gut microbiota dysbiosis affects the development of the gut microenvironment, as well as the onset of various external systemic diseases and metabolic syndromes. Cyclophosphamide (CTX) is a commonly used chemotherapeutic drug that suppresses the host immune system, intestinal mucosa inflammation, and dysbiosis of the intestinal flora. Immunomodulators are necessary to enhance the immune system and prevent homeostasis disbalance and cytotoxicity caused by CTX. In this study, shrimp peptide hydrolysate (SPH) was evaluated for immunomodulation, intestinal integration, and microbiota in CTX-induced immunosuppressed mice. It was observed that SPH would significantly restore goblet cells and intestinal mucosa integrity, modulate the immune system, and increase relative expression of mRNA and tight-junction associated proteins (Occludin, Zo-1, Claudin-1, and Mucin-2). It also improved gut flora and restored the intestinal microbiota ecological balance by removing harmful microbes of various taxonomic groups. This would also increase the immune organs index, serum levels of cytokines (IFN-ϒ, IL1β, TNF-α, IL-6), and immunoglobin levels (IgA, IgM). The Firmicutes/Bacteroidetes proportion was decreased in CTX-induced mice. Finally, SPH would be recommended as a functional food source with a modulatory effect not only on intestinal microbiota, but also as a potential health-promoting immune function regulator.

## 1. Introduction

The gut microbiome is a diversified community of trillions of bacteria (microorganisms) that live in the mammalian body and perform a variety of physiological functions in the host’s gastrointestinal tract [1,2]. The host and the microbiota have common associations in a micro-ecosystem in the digestive tract. The microbiota is pivotal in regulating host metabolism, i.e., digesting the carbohydrate to supply the nutrients for maintenance of metabolic system, vitamin synthesis, maintain physiology, protect colonization of the pathogen by shielding the harmful microbe’s invasion [3,4,5]. The intestinal barrier system consists of four layers that work together to keep the gastrointestinal tract in a state of homeostasis: immunological, mechanical, biological, and chemical barriers [6,7]. The mechanical barrier is composed of different types of cells: goblet cells, absorption cells, intestinal stem cells, endocrine cells, and Paneth cells, all of which collaborate to maintain the function and structure of the intestine [8]. Mucin-2 (Muc-2) is a component of chemical barriers, formed as an exudate of goblet cells and adheres to the surface of epithelial cells to prevent pathogens from attacking the mechanical barriers of the intestine [8,9]. In the intestine, the microbiota stimulates cellular proliferation, mucus production, villus thickening, vascularization, epithelial junction maintenance, and mucosal surface widening [10,11]. Gut microbiota dysbiosis plays a role in the development of the gut microenvironment, as well as the onset of various external systemic diseases and metabolic syndromes [5,12,13]. Nonetheless, commensal microbes play a vital role in gastrointestinal secretions, immunological response, and food metabolism [4,5,14].

In recent years, there has been a general understanding of the gut microbiota, with the rational use of probiotics as a functional food being well-established [2,15,16]. Pathogens are known to cause infections and are strongly associated with the development or enhancement of disease. While commensal gut microbiota modifies both genetic and epigenetic factors and modulates host health directly or indirectly [8,12], there is an established between immunity, the mucous membrane immune system, and the gut microbiome. Intestinal flora imbalance results in downregulation of immunological response, which in turn impacts immune system development, altering the immune response of distant mucous membrane tissue [13,14]. The nature of the intestinal flora is altered by an abnormal immune system, which in turn influences the immune response of the distant mucosa, leading to immune imbalance [14]. The host responds to the metabolites produced by microbes, altering the growth of immune cells and resulting in the formation of an immunological response at the limits of the body [13].

Since 1958, CTX has been one of the most used chemotherapeutic drugs to treat autoimmune disease and cancer, despite its dominating adverse effects of destroying and interfering with cells’ DNA, folic acid, and thus causing immunosuppression [7,15,16,17]. A high dose of CTX may affect not only tumor cells, but also lead to downregulating the immune system while increasing intestinal mucosa inflammation, dysbiosis of the intestinal flora, and increased permeability of the intestine. All of them resulting in secondary infection by potential pathogens [7,18,19]. As a result, immunomodulators are important to stimulate the immune system to reduce the adverse effects of CTX and to prevent homeostasis disbalance and cytotoxicity [18,20].

A bioactive peptide is a group of polypeptides that produce in vitro enzymatic hydrolysis and fermentation of various marine or food resources employing appropriate proteolytic enzymes [17,21], structurally contain more than 2–20 residues of amino acid, due to hydrophobic characteristics and small size, bioactive compounds are easier to absorb [19]. Marine species contain physiologically bioactive components for active pharmaceutical ingredients and are an important compound for human nutrition. Marine peptides, particularly, have attracted a lot of interest because of their potential for improving health and lowering illness risk [22,23,24]. Peptides may play a significant role in the intestine by regulating digestive enzymes, stabilizing the intestinal epithelial tight junctions, and modulating dietary absorption [25,26]. Peptide hydrolysate from marine sources is safe, physiologically beneficial, improves immunity, and performs a variety of functions, i.e., anticancer, antibacterial, antiviral, antihypertensive, antithrombotic, hormonal, and cholesterol-lowering effects [20,22,23,27]. It has been reported that shark-derived hydrolysate upregulates the cytokines production and protects intestinal epithelial [24]. Oyster peptides stimulate cytokines, modify gut microbiota, and ameliorate intestinal damage [15].

However, the effect of shrimp peptide hydrolysate (SPH) on intestinal microbiota and mucosal integrity is less studied and their functional activity is not known. Therefore, the current study investigates the effect of SPH on immunomodulation, intestinal integration, intestinal microbiota in cyclophosphamide induces immunosuppressed mice.

## 2. Results

### 2.1. Protein Concentration of SPH

The concentration of SPH was determined by the Bradford method. The hydrolysate concentration increases hourly, and chymotrypsin (1%) concentration gives maximum yields of protein hydrolysate (Figure S1). The tris-tricine SDS gel electrophoresis analysis of SPH revealed that these proteins were of low molecular mass peptides, i.e., <10 kDa shown in Figure S2.

### 2.2. Molecular Mass Distribution of SPH

**Figure 1:**
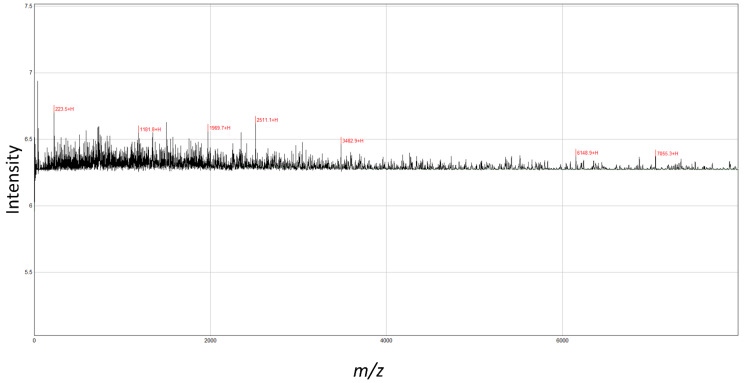
MALDI-TOF-MS analyses of peptides from shrimp protein hydrolysate. The mass spectrum was obtained from the peptide sample. Peaks with *m*/*z* values indicate the identified peaks.

The MALDI-TOF-MS analysis revealed seven peaks in SPH (Figure 1). The analyzed molecular mass distribution was 0–8000 *m*/*z*. Five high-intensity peaks (3482.9, 2511.1, 1969.7, 1181.8, 223.5) were identified at <4000 m/z as major peptides.

### 2.3. SPH Ameliorative Effect on Immunosuppressed BALB/c Mice

**Figure 2:**
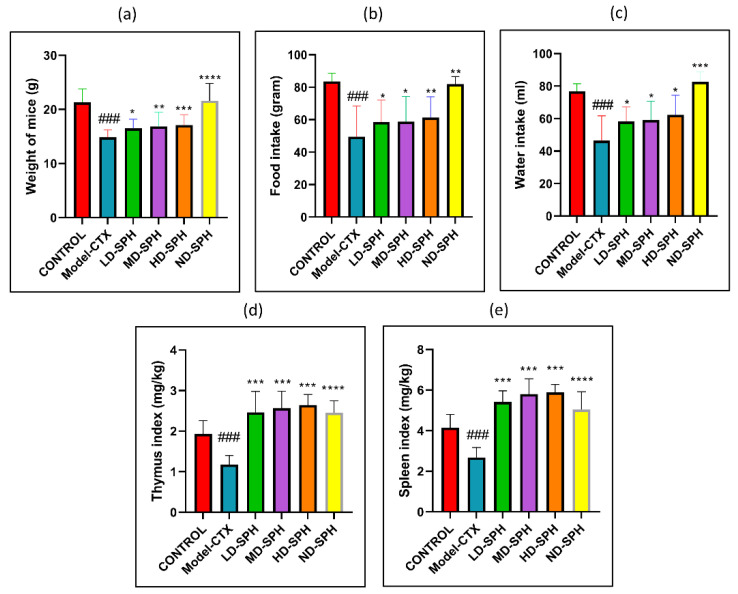
SPH effect on body weight, food and water intake, immune organ indices, and on proliferation response of splenocyte in CTX treated mice (6 groups, *n* = 10.) (**a**) Bodyweight. (**b**) Water intake. (**c**) Food Intake. (**d**) Spleen index. (**e**) Thymus index. ### *p* < 0.001 comparison to the Normal control group. * *p* < 0.05, ** *p* < 0.01, *** *p* < 0.001, and **** *p* < 0.0001, comparison to the Model-CTX group.

CTX administration was significantly decreased food and water intake, bodyweight in the Model-CTX group, as shown in Figure 2a–c. In SPH treatment groups, mice had significantly improved body weight and food/water intake as compared with the model group. The spleen and thymus indices of the control group were significantly higher (*p* < 0.05) than the model group. Spleen and thymus indices of SPH treatment groups were significant compared with the model group (*p* < 0.05) while no significant differences were noted between the SPH treatment groups and the control groups (Figure 2d,e).

### 2.4. SPH Effect in Immunosuppressed Mice on Cytokine Levels

**Figure 3:**
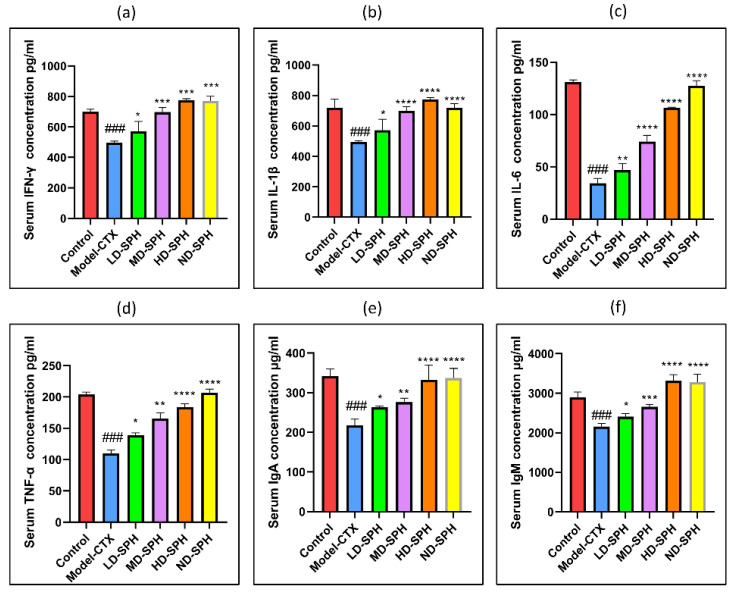
Effects of SPH on serum cytokines (**a**) IFN-γ, (**b**) IL-1β, (**c**) IL-6, (**d**) TNF-α, and immunoglobulin concentration (**e**) IgA, and (**f**) IgM. ### *p* < 0.001 comparison to the Normal control group. * *p* < 0.05, ** *p* < 0.01, *** *p* < 0.001, and **** *p* < 0.0001, comparison to the Model-CTX group.

**Figure 4:**
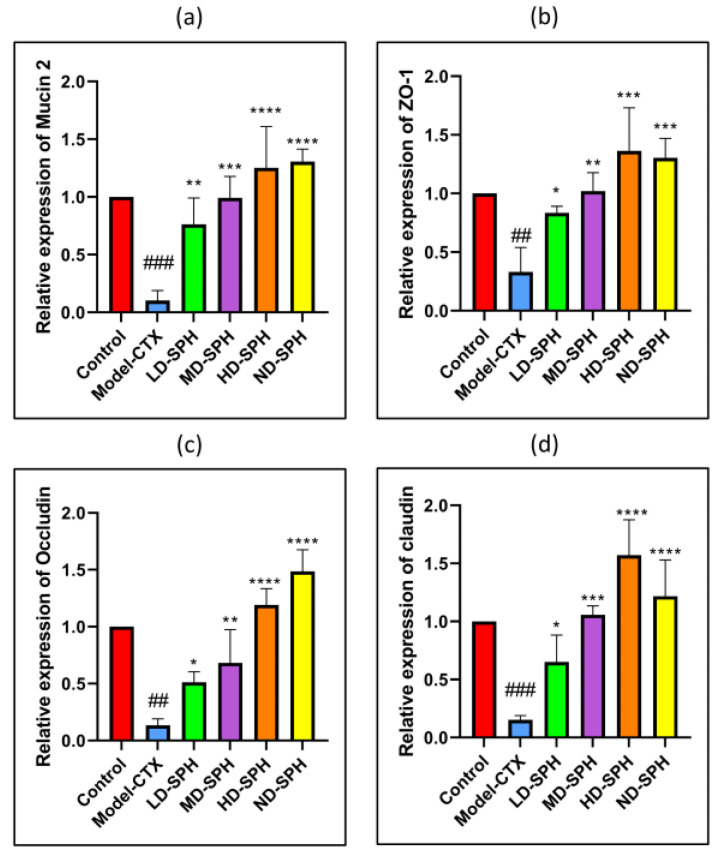
Relative expression of mRNA in colon tissues: (**a**) Mucin-2, (**b**) Zo-1, (**c**) Occludin, and (**d**) claudin-1, mRNA levels were standardized against Beta-actin expression, and as shown the mean ± SD fold increase compared with the control group. ### *p* < 0.001 comparison to the Normal control group. * *p* < 0.05, ** *p* < 0.01, *** *p* < 0.001, and **** *p* < 0.0001, comparison to the Model-CTX group.

The serum concentration of cytokines, i.e., TNF-α, IFN-ϒ, IL-1β, IL-6, and immunoglobulins (IgA, IgM) in the model-CTX group were significantly lower (*p* < 0.05) than the control group. Serum cytokine (TNF-α, IFN-ϒ, IL-1β, IL-6) and immunoglobulins (IgA and IgM) concentration was significantly increased at post-treatment of SPH (Figure 3a–f). Colonic tissue mRNA expression of Mucin-2, Occludin, Zo-1, and claudin-1 was decreased in the Model-CTX group while SPH treatment groups showed a significant increase (Figure 4a–d).

### 2.5. Effects of SPH on Mouse Spleen, Thymus, and Colon Histomorphology

**Figure 5:**
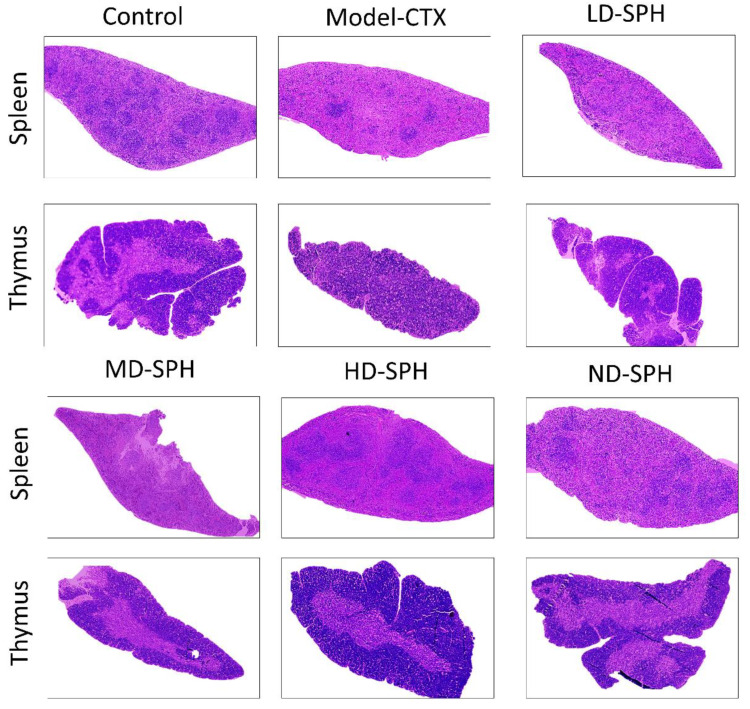
SPH impacts on spleen and thymus tissues histology. Magnification 10×, Scale bar: 100 µm.

The histomorphological changes in the spleen and thymus as well as the effects of SPH are shown in Figure 5. The morphology of the spleen’s white and red pulp was observed normal through H&E staining in the Normal control group. The lymphoid nodules were clear, with lymphocytes clustered together. The spleens in the Model CTX group had a disrupted structure, with an unclear margin between the white and red pulps. Furthermore, there is an ambiguous border between the medulla and the thymic cortex. The thymocyte estimation was decreased in the Model CTX group. While the SPH treatment groups (LD.SPH, MD.SPH, HD.SPH, and ND.SPH) have had similar results as a control group, with tightly organized thymocytes comprising a clear nucleus and fewer intracellular spaces in a dose-dependent manner. Histomorphological findings showed that SPH reserved the induced CTX impairment in the thymus and spleen in a dose-dependent manner.

**Figure 6:**
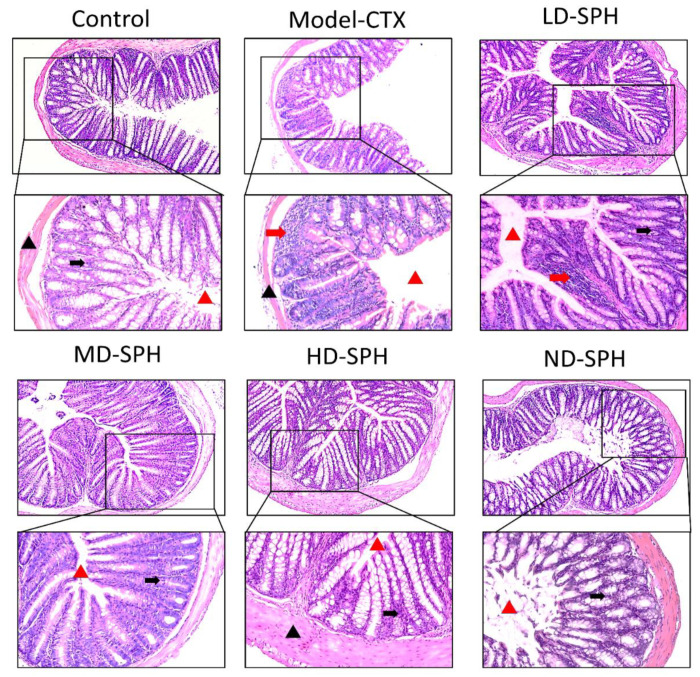
Effect of SPH on colon histologic modifications in mice with dysregulation caused by CTX. Colon sections stained with hematoxylin and eosin (H&E). Inflammatory cells (represented by red arrows), the mucosal space (represented by a red arrowhead), epithelial cells (represented by black arrows), and the epithelial surface (represented by black arrowhead). Magnification 20×, scale bar: 100 µm.

**Figure 7:**
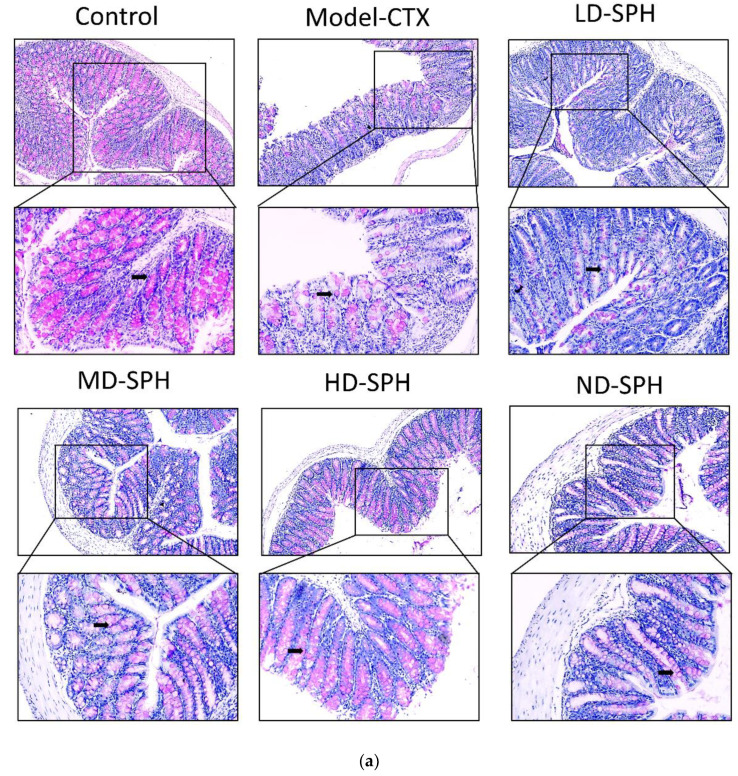

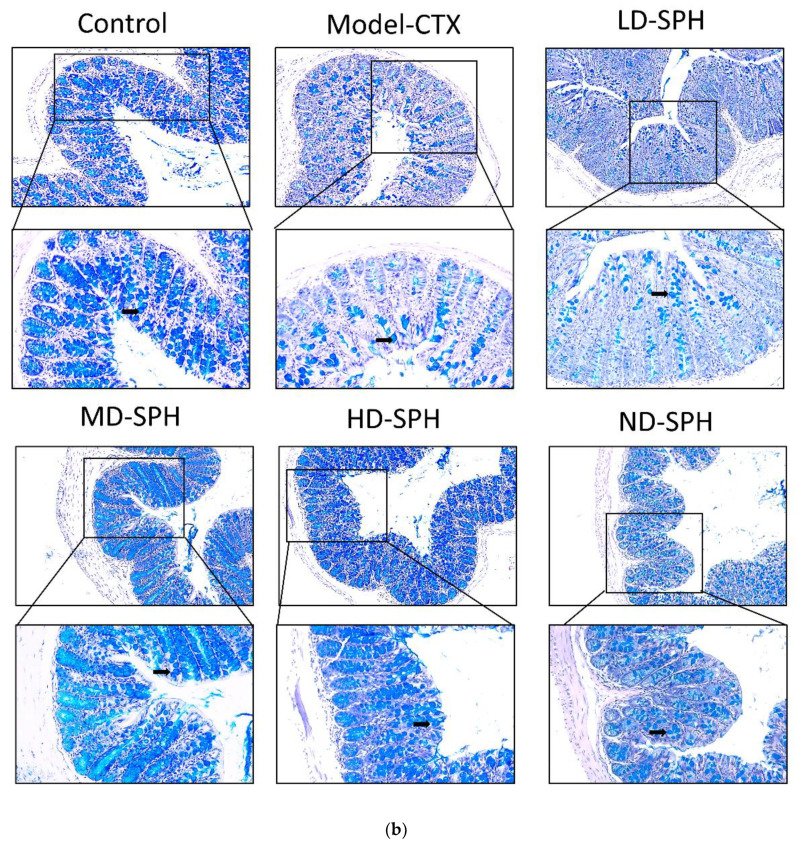
SPH treatment replenished goblet cells and enhanced mucin secretion in CTX-induced mice. (**a**). PAS staining (**b**). Images of colon sections are represented by AB-PAS staining. In each group, the number of goblet cells and mucin production were measured. Control mice revealed plenty of goblet cells with higher mucin expression (black arrows). In the CTX group, goblet cells were depleted completely. In SPH treated groups the number of goblet cells improved and the mucin expression is enhanced particularly at a higher dose. Magnification (upper 100×) (lower 200×).

**Figure 8:**
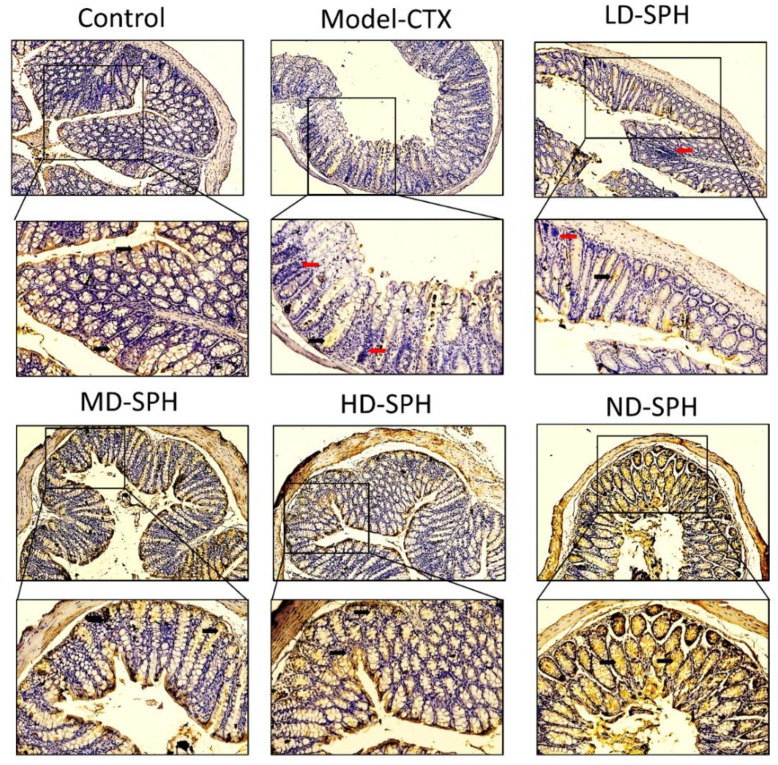
Mucin-2 immunohistochemistry staining in the colon of various groups Mucin expression (demonstrated by black arrows) and inflammatory cells are shown (demonstrated by red arrows), Original magnification 10×, 20×, scale bar: 100 µm.

**Figure 9:**
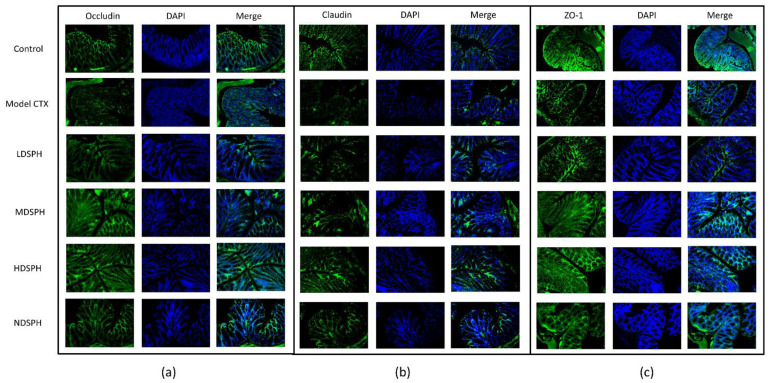
Immunofluorescent staining of tight junction proteins in the colonic epithelium (**a**) Occludin (**b**) Claudin (**c**) ZO-1. Original magnification 20×, scale bar: 100 µm.

The histomorphology of the colon shows that permeability increased after the administration of CTX, villi breakage, elongation of shallow crypts, loss in goblet cells as shown in Figure 6. The goblet cells proportion was evaluated in the colon after cyclophosphamide treatment (Figure 7a,b). The Model-CTX had a significantly lower relative number of goblet cells when compared with the control group. Periodic-acid Schiff (PAS) was utilized to stain neutral glycoprotein or mucus (purple-stained) containing goblet cells in the colon tissues. PAS and Alcian blue periodic-acid Schiff (AB-PAS) staining revealed that the glycoprotein content was significantly decreased in the Model-CTX group compared with the control group. Moreover, SPH treatment increased and recovered the enormous amount of goblet cells and mucin expression in LD.SPH, MD.SPH, HD.SPH, and ND.SPH in a dose-dependent manner. The expression of tight junction proteins in the colonic epithelium is affected by morphological changes. To further understand the impact of CTX on a mucosal barrier, Mucin2, Occludin, Claudin, and ZO-1, three of the most well-studied tight junction proteins were analyzed. Treatment with CTX reduced the expression of Mucin2, Occludin, Claudin, and ZO-1, while SPH treatment improved the interstitial barrier and increased the expression of tight junction protein as depicted in Figure 8 and Figure 9.

### 2.6. SPH Modulates the Gut Microbiota Composition and Metabolic Functions Profile

**Figure 10:**
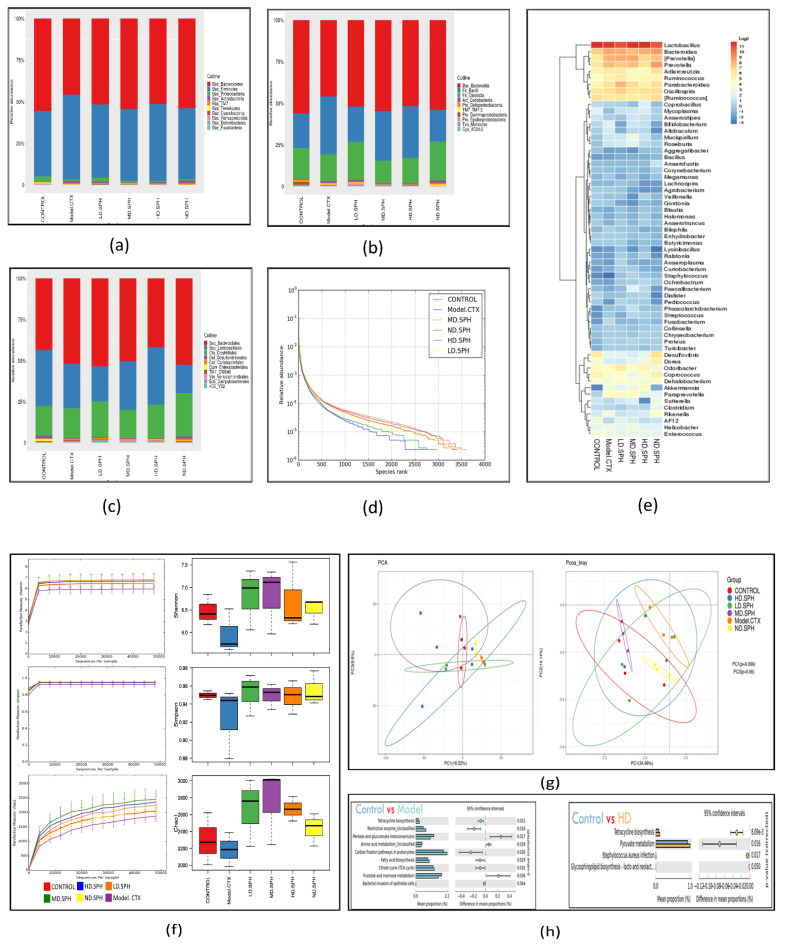
SPH’s impacts on the gastrointestinal microbiota at the molecular basis. (**a**–**c**) Individual samples at the taxonomic levels of phylum, class, and family. (**d**) A rank abundance curve depicts the richness and evenness of the species. The x- and y-axes depict the number of operational taxonomic units based on the distribution, abundance, and abundance of each species (OTUs). (**e**) Hierarchical clustering of gut microbiome via heat map analysis of the highly characterized bacterial taxa genus level. (**f**) Rarefaction curves depicting species diversity and abundance. For each treatment group, the Shannon, Simpson, choa1 alpha coefficient depicts the microbial richness and diversity. (**g**) The proportion of the mice stool specimens predicated on the evolutionary appearance of their intestinal bacteria is shown in a principal coordinate analysis (PCoA) plot with Bray–Curtis dissimilarity. Every specimen is depicted by a dot in the graph, with various colors representing a diverse experimental group (*n* = 3). The deviation between the observations is represented by the range among each other; the nearer the specimens are, the more differently compared they are. (**h**) Analysis of functional genes associated pathways in the control, Model-CTX, and treated groups.

The pyrosequencing examination of 16S rRNA was used to analyze after splicing and filtering dataset of good coverage clean 3,789,943 tags was obtained from fecal samples of all groups. The data was statistically analyzed after operational taxonomic units (OTUs) were clustered with a 97 percent similarity level. The taxonomic abundance of the intestinal microbiota was investigated at the phylum and genus level for the specific changes induced by CTX and SPH in all groups. *Bacteroidetes* and *Firmicutes* were the largest bacterial group at the phylum level (90%) as shown in Figure 10a. The next most prevalent phyla were *Proteobacteria* and *Actinobacteria.* In comparison to the control group, the CTX group demonstrated a rise in *Firmicutes* (50.90%), as well as a decrease in *Bacteroidetes* (45.70%). The CTX-induced dysbiosis in the gut microbiota was partially reversed after treatment with SPH shown In Table 1.

Bacteria at the taxonomic level of class and family were observed (Figure 10b,c). In comparison to the normal control group, the CTX group had an abundance of class; *Bacilli* (34.7%), *Coriobacteriia* (1.1%), and *Erysipelotrichi* (0.30%), while in the normal group, the abundance of class; *Bacteroides* (55.4%) and *Clostridia* (18.4%) are greater than CTX group. The abundance of fecal microbiota at family level in normal group *S24-7*(34.1%), *Prevotellaceae* (8.6%), *Lachnospiraceae* (4.9%), *Rikenellaceae* (3.5%), and in the CTX group, the greater level of *Bacteroidaceae* (7.5%), *Paraprevotellaceae* (5.7%), *Ruminococcaceae* (3.2%), *Lactobacillaceae* (34.7%), on the other hand, the CTX-induced changes were partially restored by SPH treatment. Next, we evaluated the relative abundance of bacterial genera through Heatmap; (Figure 10e) *Lactobacillus* (CTX 27.04 % vs. Control 33.83%), *Bacteroides* (CTX 5.65% vs. Control 1.6%), *Prevotella* (CTX 8.20% vs. Control 3.40%), after HD.SPH, MD.SPH, and LD.SPH in all treatment groups effectively restored the proportion of all affected phyla, demonstrating the clinical importance of SPH in the restoration of gut dysbiosis.

The community richness and diversity of each treatment group were described through a rank abundance and a rarefaction curve. The species abundance and evenness in, respectively, all treatment groups were explicitly represented by the rank abundance curve (Figure 10d), where the width of the curve and the horizontal direction represented the species richness and abundance, separately. The species richness is high in the normal and all treatment, groups compared with the CTX group. Alpha diversity reflects the richness and variation of the intestinal bacteria. The Chao1 and Ace indices are frequently used to evaluate the percentage of bacterial richness in various groups, whereas the Simpson and Shannon indices are used to assess the diversity of intestinal flora. There is no significant difference in bacterial richness and alpha diversity in separate groups analyzed with Chao1 and Ace indices, and the Shannon and Simpson indices. In Figure 10f, the rarefaction curve shows species diversity and abundance that reflects the analysis of several sequences. Shannon, Simpson, and the observed species show higher diversity and richness in the control and all treatment groups as compared to the CTX group.

The beta-diversity analysis revealed the dissimilarity or similarity of samples in species compositions shown in Figure 10g. The OTU abundance data and the Bray–Curtis inter-sample distance matrix were used for principal coordinates analysis (PCoA), and perform principal component analysis (PCA), shows apparent intestinal microbiota clustering for each group. Several parameters, including Pearson, unweighted UniFrac, weighted UniFrac, Bray–Curtis, and permutational multivariate analysis of variance (PERMANOVA), are used to measure distance and compare differences between groups in beta diversity. According to distance matrices, the intestinal microbiota of CTX-treated mice differs in species composition and clusters far from the NC group, whereas the microbiota of treatment groups is much similar, and clusters close to the NC group.

PICRUSt 1.1.0 predicts microbial ecological functions based on 16S sequences against the spices in the Greengenes database using ortholog, Cog, and KEGG pathways. Statistical Analysis of Metagenomic Profile (STAMP) (version 2.1.3) was used to further assess outcomes. In CTX treated mice and control group 6 KEEG pathways show a significant variation detected in the pairwise abundance comparisons (Figure 10h). The most enriched metabolic pathways among these were replication, repair and recombination proteins, and restriction enzymes. Bacterial invasion of epithelial cells, pyruvate metabolism, ribosome biogenesis, tetracycline biosynthesis, carbon fixation pathways in prokaryotes, TCA cycle, fatty acid biosynthesis, energy production and depleted (Carbohydrate transport and metabolism. Amino acid metabolism, pentose and glucuronate interconversions, fructose and mannose metabolism, pentose phosphate pathway, RNA processing, modification, etc.) alleyways related to those of the NC group. KEGG pathways were altered in different groups, proving that SPH therapy can enhance immunity by balancing the gut microbiota’s metabolism.

## 3. Discussion

The intestine is the largest digestive, absorbing, and immune organ, and gut intestinal microbiota and immunity continuously interact to enhance immune intestinal integrity and homeostasis [14,28]. Gut immune homeostasis is composed of epithelium, lamina propria, and Peyer’s patches, which are the first barrier against damages [14,15]. The imbalance in intestinal/gut homeostasis leads to dysbiosis of gut microbial communities, and hence, will trigger a variety of diseases and abnormal immune responses [29]. Cyclophosphamide is a potent immunosuppressive agent, and a widely used anticancer drug, which may cause complications by the disruption of the mucosal barrier, and immune function that reduces the intestinal tight junctions, adherent junctions and enhances the potentially pathogenic bacteria [30,31]. Therefore, in this study, the effect of SPH on CTX induced mice were investigated to evaluate the gut intestinal integrity, gut microbiota, and immunomodulation. Factors; enzyme, PH, time, temperature, and solid–liquid ratio affected peptide extraction concentration from marine recourses. The aforementioned factors were assessed for improved product quality [32,33]. The enzymatic hydrolysis was affected by chymotrypsin (1%) at 50 °C. The SPH extracts isolated from shrimps have identified five low molecular weight peptides with MADIL-TOF-MS. Previous studies have shown that peptides fractions < 6 kDa have better immunomodulatory activity [34].

The thymus and spleen are the key component of the immune organ in the immune system and have an imperative role in nonspecific immunity [35]. Immune cell proliferation, differentiation, and activation lead to an increase in the weight of the immune organs [36,37,38,39], and vice versa represents the decreased activity of immune functions [40,41,42,43]. After the administration of CTX, the body weight and immune organ index of mice was significantly decreased and at post-treatment, with SPH the mice’s body weight, spleen, and thymus were significantly increased (*p* < 0.05) in all SPH treatment groups as compared with model group. Zhang and Wang reported similarly and stated that peptide hydrolysate increases body weight and organ index [40,44]. The spleen and thymus, which are crucial organs in the immunological response, have been reported to be damaged when CTX is administered [41]. The micromorphological observation shows that spleens displayed a disrupted structure in the CTX Model group, with an unclear margin between the white and red pulp, lymphocyte count decreased, and reticular cells increased. Thymus shows uncertain margin, low thymocyte. These changes are due to atrophy of the immune organ and as an indication of immunosuppression. After treatment with SPH, the found changes were deprived and the number of lymphocytes, thymocytes increased, the pulps’ margin was clear, and spleen and thymus tissues were regular.

The second line of defense of the gut intestinal barrier is epithelial cells, which play a direct role in the gut’s immunological maintenance. Epithelial cells play an important role not only in the response of pathogens but also in the transmission of the signal to the intestinal immune system through the release of cytokines and inflammatory mediators [45]. Cytokines are small soluble glycoproteins derived from a variety of cells that stimulate differentiation, cell division, and multiplication through plasma receptor molecules in interacting cells [42]. Cytokines are important mediators and regulators of the immune response, and their secretion level may reflect the body’s immune function [15]. In a CTX-induced immunosuppressed model, a peptide from yak collagen hydrolysates significantly increased serum levels of cytokines (IL-2, TNF-α, IFN-ϒ), immunoglobin (IgA, IgG), and improved humoral and cellular immune responses [43]. CTX-treated mice showed a substantial reduction in serum cytokine, similar to prior observations [19]. After the administration of SPH, cytokines expression TNF- α, IFN-γ, IL-1β, IL-6, and immunoglobulin IgA, IgM was found to be elevated in our studies. In previous studies, oyster peptide stimulated cytokines such as IL-2, IFN-ϒ, IL-4, and IL-10 [15], and Nibea japonica peptide significantly increased cytokine secretion such as IL-2, IFN-ϒ, and TNF-α [46].

Intestinal epithelial cells keep normal intestinal permeability, intact structure, and protect against pathogens or hazardous chemicals [47]. Goblet cells and tight junction protein play a key role to maintain intact intestinal structural, intestinal barrier function, regulation of gut permeability, and maintaining the epithelial cell barrier. It produces mucus, which effectively helps to protect invading pathogens and hence, maintenance of gut health [48]. Moreover, goblet cells produce multiple mucins that play an important role to maintain the mucosal barrier and protect against microbes [48,49]. Claudin and occludin are transmembrane proteins that interact with the extracellular environment and connect adjacent cells, ZO-1 connects claudin and occludin which may help to keep tight junctions intact [50,51]. In the CTX model group staining results showed intestinal and mucosal barrier disruption, and a decrease in the goblet cells and tight junction proteins. The SPH restores the damaged mucosal integrity caused by CTX through restore goblet cell populations and increasing the production of tight junction proteins. These results were consistent with other studies treated with cyclophosphamide [15,28].

Nutraceuticals not only contribute to a better gut structure, but also improve the host’s functional activities, such as absorption and immune system [52]. The feces contain the most microbiota along with the gastrointestinal system contents, would easy to collect, is far less disruptive. It presents alterations in gut microbiota associated with health and disease [31]. CTX has shown arbitrarily dysregulation of immune cells and intestinal mucosa cells, resulting in enteritis by increasing permeability, reducing the intestinal barrier immune system, and changing the microbial populations of the small intestine [21,53,54]. Intestinal flora (microbiota) composition was compared between the normal and CTX treatment group at the phylum show the abundance of *Firmicutes*, and less abundant *Bacteroidetes* in the CTX group, these results were consistent with previous studies [18,21,54,55]. In addition, immune function dysbiosis was observed in immunosuppressed mice with an abundance of *Bacteroides* [56]. The fecal microbiota of chemotherapeutic induced mice, *Bacilli*, *Erysipelotrichi*, *TM7-3*, *Coriobacteriia* were increased while *Bacteroides*, *Clostridia*, *Deltaproteobacteria* were decreased, Xu. X and Gu. S. reported similarly [31,57]. The healthy gut microbiota of mice has a well-balanced composition of microorganisms of several groups patterns were connected to low-grade intestinal inflammation irrespectively of mouse strain [58,59]. These data demonstrated associations between CTX-induced immune responses and variations in bacterial abundance in diverse bacterial groupings.

The Chao1 and Ace indices metrics were assessed to compare the number of OTU in a sample; a high value provides higher biodiversity of beneficial gut flora. Chao 1 and Ace indices were the most common indicators to predict the amount of group abundance, whereas Shannon and Simpson’s indexes were the most common indicators of group biodiversity [60]. Alpha diversity reduces microbial richness and diversity after administration of CTX and after the administration of SPH, the richness and diversity of the spices reversed and enhanced in the treatment group shown in Figure 10f. Beta diversity indices indicate the dissimilarities among different treatments which exhibit that the CTX group where deviated as compared to the control group, whereas the control group and SPH treated groups were closely clustered shows more similarities between groups. Previous studies also show that the richness and diversity of microbiota decrease after administration of CTX [15,31]. In our study, metagenomic functional analysis prediction revealed a difference between the control and CTX groups. The metabolic and cellular pathways are linked to the higher and lower abundance of certain genes’ carbohydrate transport and metabolism, amino acid metabolism, fructose and mannose metabolism, pentose and glucuronate interconversions, RNA processing, and modification. Pentose phosphate pathways all have low abundance in the CTX group.

The absence of carbohydrate metabolism, as well as RNA processing and modification, has an effect on organism physiological processes, the host immune system, and nutrient absorption. Changes in carbohydrate metabolism have previously been connected to the composition and metabolism of gut-associated intestinal bacteria [53,61]. In our research, we observed that SPH may improve carbohydrate metabolism induced by the CTX. Ultimately, we revealed that SPH improved nutrient absorption and energy utilization while also maintaining gut integrity, intestinal flora composition, preventing pathogens and their metabolites, and remodeling gut metabolome roles in immunocompromised mice, implying that SPH may play an immunologic protective role.

## 4. Materials and Methods

The shrimps (*Penaeus chinensis*) were purchased from the supermarket (Lvshunkou. Loaning, Dalian, China). RIPA lysis buffer was bought from Beyotime Biotechnology (Shanghai, China). Chymotrypsin was purchased from Sigma-Aldrich Trading Co., Ltd., (Shanghai, China). A protein quantification assay kit (BCA) was Purchased from Jiancheng Bioengineering Institute (Nanjing, China). ELISA assay kits for TNF-α, IFN-γ, IL-1β, IL-6, IgA, IgM, Rabbit antibodies were supplied by Jiangsu Mmmiology biological Co., Ltd., (Jiangsu, China); others all reagents were of analytical grade.

### 4.1. Preparation of SPH from Shrimp

SPH was prepared using the hydrolysis process approach with a few tweaks, as discussed earlier. Whole shrimps were mixed in a grinder and then washed in the double volume of distilled water at 98 °C for one hour. After wash, the smashed shrimp residues were filtered through meshes (150 μm) and combined with two volumes of distilled water before being digested at 50 °C for six hours with a 1 percent (*w*/*w*) chymotrypsin enzyme. The enzymes activity was then halted for 15 min by heating to 98 degrees Celsius. The digested lysates were centrifuged for 20 min at 14,000 rpm to extract the supernatant, which was used to make shrimp hydrolysate. The Bradford technique was used to measure the SPH concentrations.

### 4.2. Molecular Mass Distribution of SPH

Shrimp peptide hydrolysate was evaluated for molecular Mass distribution using Proteomics Analyzer for matrix-assisted laser desorption ionization-time of flight mass spectrometry (MALDI-TOF-MS) (Bruker, Germany).

### 4.3. Animals and Experimental Design

Animal Laboratory and Safety Research provided a total of 60 disease-free BALB/c mice (3 to 4 weeks old) with a bodyweight of 18 ± 2 g. The experimental and Animal Ethics Committee of Dalian Medical University approved the animal studies, which followed national and institutional guidelines for experimental animal handling. After one week of acclimating to their new surroundings, the mice were randomly divided into six groups (*n* = 10) based on their body weight. Normal Control, (NC), CTX model group, High dose (HD.SPH 400 mg/kg), Medium dose (MD.SPH 200 mg/kg), Low dose (LD.SPH 100 mg/kg), and Normal Control + Dose (ND.SPH 200 mg/kg). The four groups of mice were administered with 80 mg/kg/day of cyclophosphamide (CTX) intraperitoneally for 5 days according to body weight, with an equal volume of PBS administered to the NC and ND groups. From the day 6 to 30, the mice in the four groups were given 100, 200, and 400 mg/kg/day of SPH by gavage. Meanwhile, the NC and Model groups are given the same amount of distilled water orally. The procedure of animal experiments is shown in Figure S3

### 4.4. Determination of Body Weight, Food/Water Intake, and Immune Organ Index

During the administration period, body weight was recorded every day, and food and water intake were measured and recorded every third day. After the mice were forfeited by cervical displacement, the immune organs, i.e., spleen and thymus were collected and weighed immediately, and the immune organ index was calculated using a formula:

Spleen or thymus index (mg/g) = weight of organ (mg)/weight of mouse (g).

### 4.5. Measurement of Serum Antibodies, Serum Cytokines Level

After the mice were sacrificed, the whole blood samples were collected and centrifuged at 4000× *g* for 5 min. The serum was transferred into a 1.5 mL tube and stored at −20 °C. Serum IgA, IgM, and cytokine concentrations (IFN-ϒ, IL-1β, TNF-α, IL-6) were analyzed through the ELISA method, according to the manufacturer’s instructions.

### 4.6. Determination of Intestine mRNA

Muc-2, ZO-1, occludin, and claudin-1 mRNA expression levels were measured. Total RNA was extracted from colonic tissue using Vazyme Total RNA Extraction Reagent (Vazyme biotech Co., Ltd.) according to the manufacturer’s instructions. The total RNA was stored at −80 °C. The complementary DNA was then transcribed using the commercial kit HiScript II Q RT SuperMix (Vazyme biotech Co., Ltd.). The ChamQ SYBR qPCR MasterMix kit was used to perform quantitative PCR in Bioer light gene 9600 analyzers (Hi-tech (Binjiang) District, Hangzhou, 310053, China). Table S1 shows the primer sequences. The real-time PCR was run at 95 °C for 5 min, then 40 cycles of 95 °C for 20 s, primer annealing temperatures at 60 °C for 30 s, and extension at 72 °C for 30 s. Each sample was tested three times, and the instrument software gene 9660 was used to calculate and analyze relative expression, as well as GraphPad prism 8 to analyze differences between groups.

### 4.7. Histopathological Examination of Thymus, Spleen, and Colon

Colon, spleen, and thymus were collected, fixed with 10% formalin, and processed. Thin sections of 5 µ were made by microtome, stained with hematoxylin and eosin staining. Microscopic examination for histological changes was observed under a microscope.

Immunohistochemistry was used to examine the levels of expression of Mucin-2 in the colon tissue. Then, 5 µm paraffin-embedded spleen tissue cut, placed into positive charge slide, and deparaffinized in xylene. Rehydrate in a series of ethanol, follow the protocol of immunohistochemical staining kits SP-9001 (Zhongshan Goldenbridge Biotechnology, Beijing, China) according to manufacturer instruction. Results were evaluated semiquantitative method each slide was observed for immunolabeled cells in five fields for three-time randomly.

Immunofluorescent staining was used to examine the levels of expression of occludin, ZO-1, and claudin in the colon. Then, 5 µm paraffin-embedded spleen tissue was cut, placed into positive charge slide, deparaffinized in xylene, and rehydrated in series of ethanol. Tissue sections were treated for 30 min in citrate buffer for antigen retrieval at 100 watts on microwave and cool down for 1 h. Then, tissue slides were added to Blocking with 3% BSA solution for 1 h. Tissues were incubated overnight at 4 °C against occludin, claudin, or ZO-1 (1:200) antibodies. After washing, tissue sections were incubated with Alexa 488-conjugated secondary antibodies for 60 min, and nucleus staining DAPI was used. Images were taken using a confocal scanning microscope.

### 4.8. Microbiota 16S rRNA Pyrosequencing

PowerMax (stool/soil) DNA isolation kit (MoBio Laboratories, Carlsbad, CA, USA) was used for total fecal microbial genomic DNA was extracted for all the samples and stored at −20 °C. DNA quality and quantity were measured using nanodrop. Microbial 16S rRNA gene V4 region PCR amplification was done by using forward primer 515F (5′- GTGCCAGCMGCCGCGGTAA-3′) and the reverse primer 806R (5′-GGACTACHVGGGTWTCTAAT-3′), and sequenced with Illumina NovaSeq6000 platform at GUHE Info technology Co., Ltd. (Hangzhou, China). QIIME software version 1.9 pipeline was used for sequence data processing, as defined before [54]. Sequencing low-quality through the following criteria [55,62] paired-end reads and operational taxonomic unit (OTU) clustering, detection of chimera’s clusters, and including dereplication using default parameters were conducted by Vsearch V2.4.4. The taxonomic unit was assigned for each dataset using Greengenes database.

### 4.9. Evolutionary Computation and Statistical Analysis

The QIIME and R programs(v3.2.0) were being used to analyze sequence data. OUT level alpha diversity indices and Beta diversity analysis were calculated using the QIIME. The data were further analyzed for ecologically relevant function, prokaryotic clades, and high-level phenotype using Statistical Analysis of Metagenomic Profiles (STAMP) software package v2.1.3 [63], FAPROTAX [64], and BugBase tool [65].

GraphPad Prism software was used for statistical analysis (v6.04). *p*-values of less than 0.05 were deemed significant. The Kruskal–Wallis test from the R stats package was used to determine whether there was a statistically significant difference among the groups based on 16S rRNA data. Mann–Whitney test was used to compare OTU counts and phenotype.

## 5. Conclusions

In this study, SPH immune modulates the effects of CTX on the gut intestine of immunosuppressed. SPH significantly increased immune organs index, reinstated the goblet cells, intestinal mucosa integrity, enhanced the serum levels of cytokines (IL-2, IFN-ϒ, IL1β, TNF-α, IL-6) and IgA, IgM, and increased the mRNA of tight-junction associated proteins (occludin, ZO-1, claudin-1, and Muc-2). SPH would also improve dysbiosis and modified the intestinal gut microbiota ecology by reducing pathogenic bacteria at various taxonomic levels. Firmicutes/Bacteroidetes proportion was reduced in CTX-induced mice. SPH can be employed as a prebiotic source with a modulatory influence on gut microbial ecology as a possible health-promoting regulator of the gut microbiota.

## Figures and Tables

**Table 1:** Different treatment groups’ percentages of bacterial phyla.

	Control	HD.SPH	LD.SPH	MD.SPH	Model. CTX	ND.SPH
*p__Bacteroidetes*	55.83%	51.47%	51.83%	54.47%	45.95%	54.12%
*p__Firmicutes*	38.95%	46.06%	43.78%	42.98%	50.72%	42.23%
*p__Actinobacteria*	0.21%	0.76%	0.79%	0.34%	1.13%	1.54%
*p__Proteobacteria*	3.22%	0.64%	2.21%	1.10%	1.03%	1.05%
*p__TM7*	0.26%	0.26%	0.41%	0.12%	0.80%	0.71%
*p__Verrucomicrobia*	0.61%	0.20%	0.09%	0.03%	0.02%	0.20%
*p__Tenericutes*	0.65%	0.19%	0.12%	0.34%	0.22%	0.10%
*p__Cyanobacteria*	0.04%	0.13%	0.49%	0.35%	0.05%	0.02%
*p__Deferribacteres*	0.11%	0.01%	0.05%	0.08%	0.04%	0.00%
*p__Fusobacteria*	0.00%	0.00%	0.00%	0.00%	0.00%	0.00%

## Data Availability

The original data for this work are available upon email request to the corresponding author.

## Supplementary Materials

The following supporting information can be downloaded online, Figure S1: Effect of enzyme different chymotrypsin concentration on SPH concentration; Figure S2: Tris-tricine gel electrophoresis; Figure S3. The animal experimental procedure was used in this research study; Table S1: PCR primers used for qPCR.
