# Supplementary material for: Effects of Shrimp Peptide Hydrolysate on Intestinal Microbiota Restoration and Immune Modulation in Cyclophosphamide-Treated Mice
Source: Molecules. 2022 Mar 6;27(5):1720. doi: 10.3390/molecules27051720 (PMC8911659; doi:10.3390/molecules27051720)
Supplement: Supplementary file 1 [file molecules-27-01720-s001.zip › molecules-1588079-supplementary.pdf]

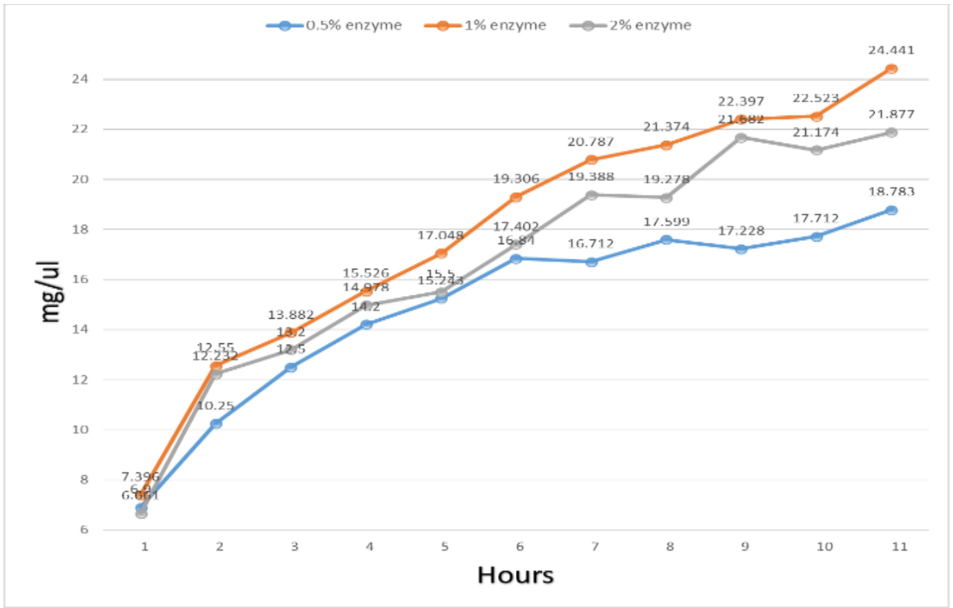

Figure S1. Effect of enzyme different chymotrypsin concentration on SPH concentration.

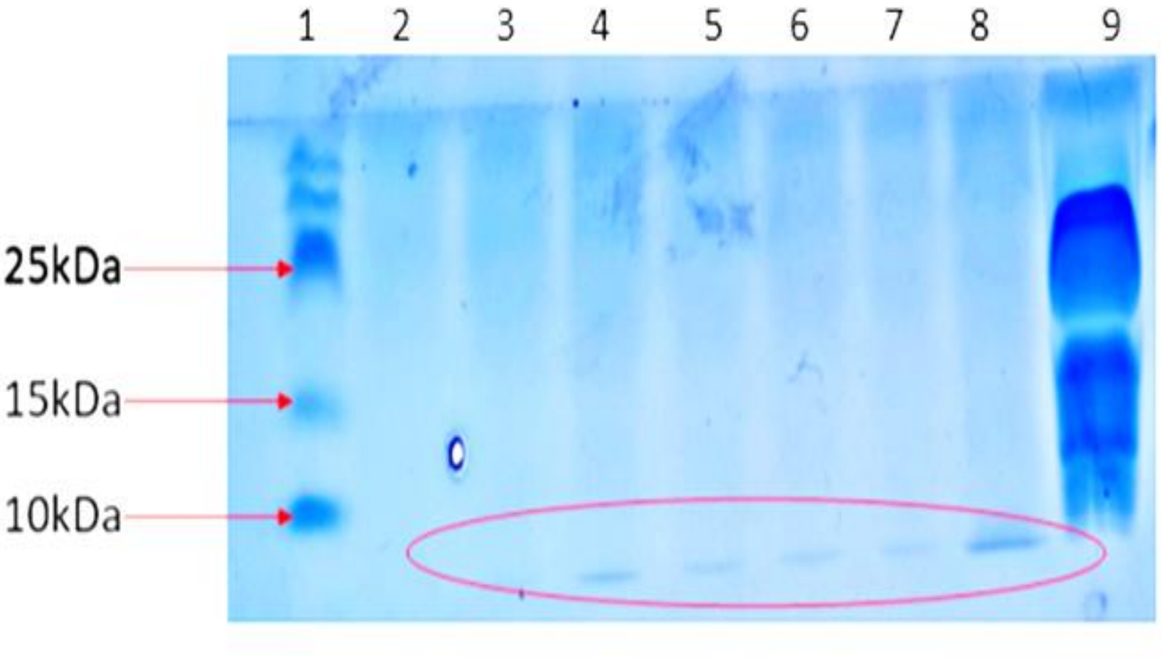

Figure S2. Tris-tricine gel electrophoresis Lane 1: marker, Lane 2 : 30min, Lane 3 : 1 hours, Lane 4 : 2 hrs , Lane 5 : 3 hrs , Lane 6 : 4 hrs , Lane 7 : 5 hrs , Lane 8 : 6 hrs , Lane 9: enzyme

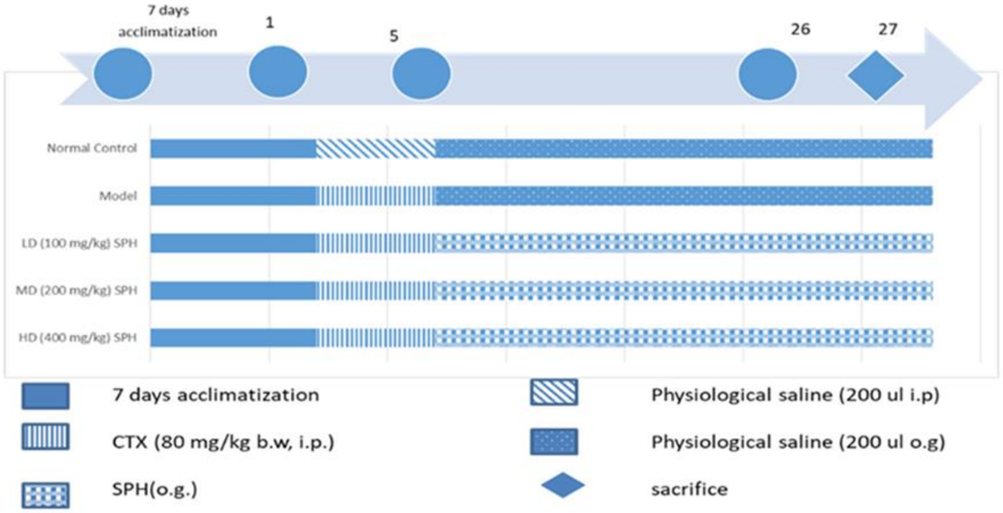

Figure S3. The animal experimental procedure was used in this research study.

b.w bodyweight o.g, oral gavage i.p intraperitoneal injection: SPH shrimp peptide hydrolysate

Table S1. PCR primers used for qPCR

Cytokines	Primer	Sequence (5'–3')
ZO-1	F	CCCTCTGATCATTCCACACA
	R	TTTAGACATGCGCTCTTCCT
Claudin-1	F	ATTGGCATGAAGTGCATGAG
	R	CCACTAATGTCGCCAGACCT
Occludin	F	CTCCAACGGCAAAGTGAATG
	R	CGGACAAGGTCAGAGGAATC
Mucin-2	F	CCCAGAAGGGACTGTGTATG
	R	TTGTGTTCGCTCTTGGTCAG
